# Serum folate receptor alpha, mesothelin and megakaryocyte potentiating factor in ovarian cancer: association to disease stage and grade and comparison to CA125 and HE4

**DOI:** 10.1186/1757-2215-6-29

**Published:** 2013-04-17

**Authors:** Daniel J O’Shannessy, Elizabeth B Somers, Leslie M Palmer, Robert P Thiel, Pankaj Oberoi, Ryan Heath, Lisa Marcucci

**Affiliations:** 1 Department of Diagnostics Development, Morphotek, Inc., 210 Welsh Pool Road, Exton, PA, USA; 2 Thiel Statistical Consultants, 45 Tram Drive, Oxford, CT, USA; 3 MesoScale Discovery, 1601 Research Boulevard, Rockville, MD, USA; 4 Frontage Laboratories, Inc., 700 Pennsylvania Drive, Exton, PA, USA

**Keywords:** Folate receptor alpha, FRA, CA125, HE4, Mesothelin, MSLN, Megakaryocyte potentiating factor, MPF, Serous ovarian cancer, Serum biomarker

## Abstract

**Background:**

Evaluate and compare the utility of serum folate receptor alpha (FRA) and megakaryocyte potentiating factor (MPF) determinations relative to serum CA125, mesothelin (MSLN) and HE4 for the diagnosis of epithelial ovarian cancer (EOC).

**Methods:**

Electrochemiluminescent assays were developed for FRA, MSLN and MPF and used to assess the levels of these biomarkers in 258 serum samples from ovarian cancer patients. Commercial assays for CA125 and HE4 were run on a subset of 176 of these samples representing the serous histology. Data was analyzed by histotype, stage and grade of disease. A comparison of the levels of the FRA, MSLN and MPF biomarkers in serum, plasma and urine was also performed in a subset of 57 patients.

**Results:**

Serum and plasma levels of FRA, MSLN and MPF were shown to be highly correlated between the two matrices. Correlations between all pairs of markers in 318 serum samples were calculated and demonstrated the highest correlation between HE4 and MPF, and the lowest between FRA and MPF. Serum levels of all markers showed a dependence on both stage and grade of disease. A multi-marker logistic regression model was developed resulting in an AUC=0.91 for diagnosis of serous ovarian cancer, a significant improvement over the AUC for any of the individual markers, including CA125 (AUC=0.84).

**Conclusions:**

FRA has significant potential as a biomarker for ovarian cancer, both as a stand-alone marker and in combination with other known markers for EOC. The lack of correlation between the various markers analyzed in the present study suggests that a panel of markers can aid in the detection and/or monitoring of this disease.

## Background

In 2012, it is estimated that 22,280 women will be diagnosed with ovarian cancer and 15,500 will die of the disease (SEER fact sheet). Ovarian cancer is considered a “silent killer” because of the absence of specific symptoms until late in the disease when 75% of the cases are diagnosed, five year survival rates are less than 30%, and a 70% recurrence rate is expected. Early diagnosis, when the cancer is confined to the ovary, can increase the 5-year survival rate to 90%. Because of the high fatality rate and relatively low prevalence of the disease, a sensitive and specific screening tool for asymptomatic women is needed. As such, effective and reliable diagnostic assays need to be highly sensitive and specific for the screening and detection of early stage ovarian cancer, especially in asymptomatic women.

CA125, a membrane-associated mucin found on the apical membrane of epithelial cells of the ocular surface, respiratory tract and female reproductive tract, is elevated in approximately 80% of women with late-stage ovarian cancer. It is the gold standard diagnostic marker to detect recurrent ovarian cancer and monitor response to treatment. However, the usefulness of CA125 as a marker cannot be extended to diagnosis as 20% of ovarian cancers do not express CA125 [1], and elevated levels are detected in only half of early stage patients. Further, CA125 is detected in many benign gynecological conditions and is particularly unreliable in detecting ovarian cancer in premenopausal women [2-5]. Additional biomarkers with high sensitivity and specificity for detecting ovarian cancer in the early stages of the disease are sought to complement CA125. Two promising markers are human epididymis protein 4 (HE4) and mesothelin (MSLN). CA125, HE4 and MSLN have been approved by the United States Food and Drug Administration (FDA) as biomarkers for recurrent ovarian cancer (CA125 and HE4) and diagnosis of mesothelioma (MSLN).

Human epididymis protein 4 (HE4), normally expressed in the epididymis, endometrial glands and respiratory tract [6,7], is up-regulated in both early and late stage ovarian cancer [6-9] including 90% of serous carcinoma, and adenocarcinomas of the lung and endometrium [10,11]. It is not expressed in mucinous carcinoma [12]. It has been widely studied as a biomarker, alone and in combination with CA125, for the diagnosis and monitoring of recurrent disease as little or no expression is observed in benign conditions [8,10,13,14]. When combined, HE4 has been shown to increase the sensitivity and specificity over CA125 alone [2,9,11,15], allowing for better detection of early stage ovarian cancer [9,15]. HE4 levels have, however, been shown to increase with age [16].

Soluble mesothelin (MSLN) has a history as a biomarker for mesothelioma diagnosis, prognosis and monitoring [17-19]. It is a differentiating antigen derived from a precursor protein that when cleaved yields Megakaryocyte Potentiating Factor (MPF), a 32 kDa excreted soluble protein [20,21], and MSLN, a 40 kDa GPI-linked glycoprotein that is also shed as a soluble form into the blood stream by frameshift mutation and proteolytic cleavage [22,23]. MSLN is hypothesized to be involved in cell adhesion and signaling [24] and to contribute to the metastasis of ovarian cancer to the peritoneum by binding CA125 [25,26]. It is highly expressed in mesothelioma, ovarian and pancreatic cancers and lung adenocarcinoma [7,23,24,27-29], but only expressed normally in mesothelial cells of the peritoneum, pericardium and pleura [27]. Like HE4, it has shown promise in the detection of early stage ovarian cancer, especially in combination with CA125 [1,30,31]. Improvements may be gained adding MSLN to a biomarker panel with CA125 and HE4 to detect early stage disease [15,31] although contradictory results have been reported [32,33]. Levels of detection of MSLN from serum and plasma for ovarian cancer were shown to be similar [34]. One issue with measuring MSLN in serum is that levels can be affected by conditions such as age, body mass index (BMI) and glomerular filtration rate [16]. Less expensive, facile screening can be achieved screening urine over serum or plasma. It is of interest, therefore, that MSLN was detected with more sensitivity in urine than serum for both early stage (42% vs. 12%, respectively) and late stage (75% and 48%, respectively) disease [35].

Although the literature is sparser than that for MSLN, a few studies have looked at MPF as a biomarker for mesothelioma [36], ovarian cancer [23] and pancreatic cancer [37]. As biomarkers in mesothelioma, MSLN and MPF have been shown to behave similarly [17-19].

Panels of biomarkers, able to cover the molecular heterogeneity of ovarian cancer [31,38], or specific to high grade serous carcinoma [12], will be the most effective way to detect early disease for fewer fatalities. To date, no panel has been identified that can achieve the sensitivity (>75%) and specificity (>99.6%) needed to meet the accepted criteria of no more than ten surgeries for every case of early stage ovarian cancer identified. The most desirable biomarkers to add to panels will be those expressed early in disease, and not expressed in normal tissue.

One such biomarker is folate receptor alpha (FRA), a glycosylphosphatidylinositol (GPI)-anchored protein involved in folate transport into cells that is expressed in breast, lung, clear cell renal, ovarian and endometrial carcinomas, and non-small cell lung adenocarcinoma [39-58]. FRA is expressed in a high percentage of serous ovarian carcinomas in all stages and grades [44,46,55,59,60], and levels of circulating FRA have been shown to be comparable between early and late stage disease [54,56]. Expression of FRA in normal tissues is restricted to the apical surfaces of some polarized epithelial cells [40].

In the present work, novel, sensitive electrochemiluminescent assays were developed for the soluble forms of FRA, MSLN and MPF and were evaluated in a large cohort of ovarian cancer patient samples. Further, the diagnostic utility of these markers was compared to CA125 and HE4 in a subset of serous ovarian cancer samples. Finally, a multi-marker logistic regression model was developed that demonstrates increased diagnostic performance relative to any single marker.

## Materials and methods

### Patient samples and controls

Samples were obtained from various commercial vendors with Institutional Review Board approvals and patient consent and were collected between 2009 and 2011. This study included serum samples from 258 ovarian cancer cases, of which 215 were serous and 47 were non-serous carcinoma (five unknown), and 60 age-matched control samples from women without disease. All non-serous ovarian cancer samples (endometrioid, mucinous, clear cell), were from women with stage I or II tumors.

In addition, serum, plasma and urine samples were collected from 57 women (37 with ovarian cancer and 20 without the disease as controls) to compare sample matrices. All serum, plasma and urine samples were collected by standard techniques and processed/frozen within 30 min of collection. All samples were stored at -80°C, and thawed and aliquotted prior to analysis. Patient demographics including date of diagnosis, histology, stage, and age (Table 1) were obtained from the suppliers.

**Table 1:** Characteristics of patients and controls

	**All histologies *n=318***		**Serous subset *n=196***	
**Variable**	**Ovarian cancer N (%)**	**Normal N (%)**	**Ovarian cancer N (%)**	**Normal N (%)**
Number of Cases	258	60	176	20
Age				
Mean	58	56	57	56
Median	57	56	56	57
Range	26-94	33-72	27-91	33-72
Race				
Caucasian/White	228 (88%)	58 (97%)	158 (90%)	19 (95%)
African American	2 (1%)	0 (0%)	2 (1%)	0 (0%)
Asian/Pacific Islander	0 (0%)	2 (3%)	0 (0%)	1 (5%)
Hispanic/Latino	1 (<1%)	0 (0%)	1 (1%)	0 (0%)
Unknown	27 (10%)	0 (0%)	15 (9%)	0 (0%)
Stage (AJCC)				
I	78 (30%)	NA	45 (26%)	NA
II	47 (18%)	NA	33 (19%)	NA
III	45 (17%)	NA	67 (38%)	NA
IV	45 (17%)	NA	31 (18%)	NA
Unknown	43 (17%)	NA	0 (0%)	NA
Grade				
Low (1)	28 (11%)	NA	17 (10%)	NA
High (2–4)	148 (57%)	NA	111 (63%)	NA
Unknown	82 (32%)	NA	48 (27%)	NA
Histology^†^				
Serous	215 (83%)	NA	176 (100%)	NA
Endometrioid	22 (9%)	NA	0 (0%)	NA
Mucinous	15 (6%)	NA	0 (0%)	NA
Clear cell	1 (<1%)	NA	0 (0%)	NA
Other^‡^	5 (2%)	NA	0 (0%)	NA

^†^Non-serous tumors are all stages I and II.

^‡^2 granulosa cell carcinomas, 1 spinocellular carcinoma, 2 undefined.

### Biomarker assays

#### Antibodies and antigens

The generation and characterization of monoclonal antibodies (MAbs) to FRA have been described previously [61]. MAbs MN and MB [62] against MSLN were purchased from Rockland Immunochemicals (Gilbertsville, PA). MAbs MPF25 and MPF49, specific for megakaryocyte potentiating factor (MPF) were obtained from Dr. Ira Pastan (Center for Cancer Research, NCI) and have been described previously [36]. All MAbs were murine IgG and purified by Protein A chromatography.

Purified, recombinant human FRA and human MSLN were the kind gift of Dr. Earl Albone (Morphotek Inc., Exton PA). MPF was prepared as a H_6_-construct and purified by metal-chelate chromatography. All antigens were demonstrated to be >98% pure by both SDS-PAGE and analytical SEC.

#### Novel electrochemiluminescence (ECL) assay

The FRA, MSLN and MPF assays all utilized ECL technology from MSD [63]. For the development of the FRA assay, 18 purified MAbs were screened pairwise and MAb pairs were selected from an unbiased screen using recombinant protein, normal pooled plasma and normal pooled urine. The final MAb pair was selected based on sensitivity, specificity, physical properties, and recognition of native protein. The selection of MAb pairs for MSLN (MN and MB) and MPF (MPF25 and MPF49) was determined based on literature. Detection MAbs were labeled with ruthenium (SULFO-TAG^TM^ NHS-Ester, Meso Scale Discovery (MSD®), Rockville, MD) and label:protein was determined according the manufacturer’s instructions. The conjugation ratio was set to 20 labels per MAb for all assays.

#### Assay protocol

Samples (serum, plasma, urine), standards or controls were added to wells of a 96-well plate previously coated with capture antibody and incubated at RT for two-hours. The Ruthenium labeled detection MAbs were diluted in assay buffer, added to washed plates and incubated for an additional two hours at RT. Plates were washed, read buffer added and signals measured using an MSD DISCOVERY WORKBENCH®. Optimal sample dilutions were: FRA (80-fold dilution of urine and a 20-fold dilution of serum and plasma), MSLN (60-fold dilution of urine and an 80-fold dilution of serum and plasma) and MPF (4-fold dilution of urine and a 20-fold dilution of serum and plasma).

CA125 and HE4 measurements were performed by Myriad RBM (Austin, TX) on a Luminex 100 instrument on 176 serum samples from women with serous ovarian tumors.

#### Statistical analyses

Pearson’s correlation coefficient was performed to determine the correlation among the various biomarkers. Pairwise comparisons of biomarker levels between normal controls and stages and grades of ovarian cancer were made using the Mann–Whitney U test. Receiver Operating Characteristic (ROC) analysis was employed to determine the performance of these markers by stage and grade of disease. ROC Area Under the Curve (AUC) calculations were based on 95% confidence intervals. Logistic regression models were developed and used to assess the performance of a panel of biomarkers relative to CA125. All comparisons were two-sided and a P-value ≤0.05 was considered statistically significant except where otherwise stated. Statistical analyses were performed in MedCalc version 12.30 (MedCalc Corp.), SPSS version 19 (IBM), GraphPad Prism version 6.00 (GraphPad Software, Inc.) and Microsoft Excel version 2010.

## Results

### ECL assay reproducibility, sensitivity and reliability

**Figure 1:**
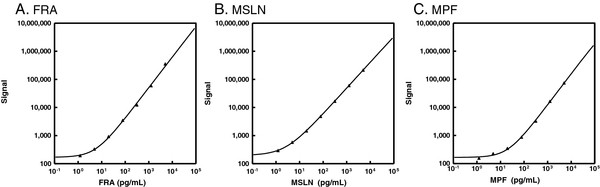
**Assay range and sensitivity representative calibrator curves. A**) FRA assay mean intraday variability (CV) ranged from 2-16% with a lower limit of detection (LLOD) of 1.22 pg/mL, **B**) MSLN assay mean CV ranged from 3-7% with an LLOD of 0.29 pg/mL and **C**) MPF assay mean CV ranged from 2-10% with an LLOD of 3.35 pg/mL.

The intraday reproducibility and sensitivity of the ECL assays for FRA, MSLN and MPF were assessed at levels between 0.01 and 5000 pg/mL (Figure 1). Mean intraday variability (CV) ranging from 2-16% for FRA, 3-7% for MSLN and 2-10% for MPF were observed and demonstrate good reproducibility for all three assays. All three assays also showed excellent sensitivity with lower limits of detection (LLOD) of 1.22, 0.29 and 3.35 pg/mL for FRA, MSLN and MPF, respectively. Representative calibrator curves are shown in Figure 1 and each assay demonstrates a wide dynamic range, potentially minimizing the need for re-assay of samples with high biomarker levels.

### Comparison of matrices: serum, plasma and urine

**Figure 2:**
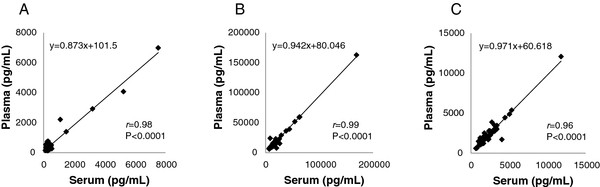
**Correlation between measurements of the various markers in plasma and serum using ECL assays for A) FRA (*r*=0.98, p<0.0001), B) MSLN (*r*=0.99, p<0.0001) and C) MPF (*r*=0.96, p<0.0001).**

To assess the most appropriate matrix for determination of the various biomarker levels, matched serum/plasma pairs from 20 normal women and 37 women with ovarian cancer were measured for FRA, MSLN and MPF using the described ECL assays. As presented in Figure 2, a high degree of concordance was observed between serum and plasma for each of the three biomarkers, with Pearson’s correlation coefficients of *r*=0.96-0.99 and slopes of 0.87-0.97, indicating that either sample type is suitable for the determination of these markers and there appears not to be a preferential distribution between either matrix. Based on these data, and the relative availability of samples, all subsequent analyses were performed using serum samples.

Of interest, however, all three markers were also detectable in urine samples from the same 57 individuals noted above, indicating that urine could serve as a diagnostic matrix in ovarian cancer.

### Diagnostic performance of FRA, MSLN and MPF in ovarian cancer cases of multiple histologies

**Figure 3:**
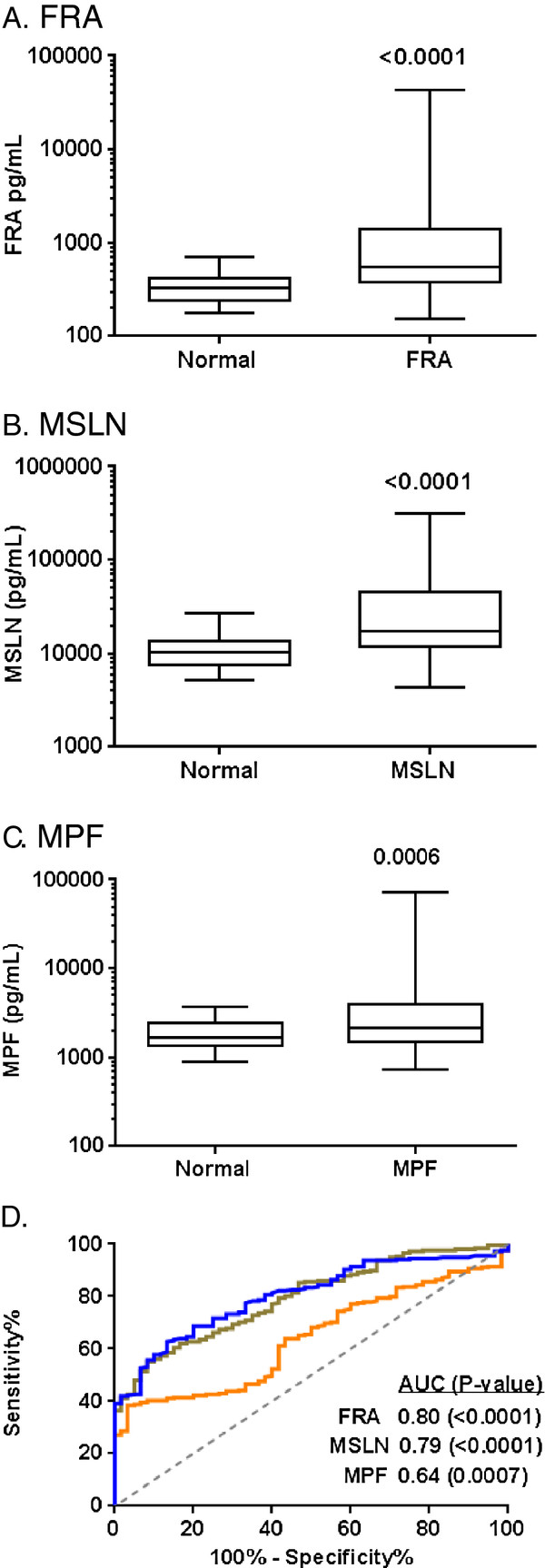
**Box plots of serum levels of A) FRA, B) MSLN and C) MPF in healthy controls and ovarian cancer patients.** Boxes indicate the 25th to 75th percentiles. The horizontal lines within the boxes are the median serum levels and the whiskers indicate the minimum and maximum values. P-values indicate statistical significance of differences between ovarian cancer cases and healthy controls. **D**) ROC (blue line, FRA; brown line, MSLN; orange line, MPF) curves showing the performance of serum biomarkers in discrimination of healthy controls and ovarian cancer patients.

Using the described ECL assays, the serum levels of FRA, MSLN and MPF were measured in samples from 258 ovarian cancers and 60 age-matched controls. Data for the ovarian cancer samples was analyzed by histotype, stage (AJCC) and grade of disease and is summarized in Table 2. Serum levels of all biomarkers differed significantly between ovarian cancer and control serum samples (Figure 3), with P values of <0.0001, <0.0001 and 0.0006 for FRA, MSLN and MPF, respectively. ROC curve analysis resulted in AUCs of 0.80 (p<0.0001) for FRA and MSLN (p<0.0001) and 0.641 (p=0.0007) for MPF (Figure 3) suggesting that FRA and MSLN have greater potential with respect to discrimination of individuals with ovarian cancer compared to normal individuals, independent of other clinical variables.

**Table 2:** Biomarker serum levels with clinicopathological findings in ovarian cancer patients

		**FRA (pg/mL)**				**MSLN (pg/mL)**				**MPF(pg/mL)**			
	**N (%)**	**Mean**	**Median**	**SD**	**Range**	**Mean**	**Median**	**SD**	**Range**	**Mean**	**Median**	**SD**	**Range**
Normal	60 (19%)	341	324	122	176-696	11225	10300	4280	5169-26891	1869	1681	618	910-3691
Ovarian Cancer	258^†^(81%)	1680	549	5870	155-42523	43002	17279	61019	4274-311565	3602	2138	5994	717-72192
Histotype^‡^													
Serous	215 (83%)	1616	586	3622	155-42523	48731	1681	62280	6322-311565	4275	2779	6446	717-72192
Endometrioid	22 (9%)	1862	434	5333	185-24843	38595	2138	64278	4274-240088	2786	1976	2887	1025-13492
Mucinous	15 (6%)	1047	497	2084	173-8475	20017	14632	19736	7672-88153	1875	1636	789	1071-4354
Stage													
I	78 (30%)	1066	412	2876	162-24843	22654	14803	44258	4274-240088	2088	1686	1826	717-9075
II	47 (18%)	977	402	2877	164-8475	30439	15891	64662	6322-225597	2509	1807	3853	832-13492
III	45 (17%)	2014	775	5103	155-42523	58945	36112	69385	6370-257344	5887	3164	8499	858-72192
IV	45 (17%)	2811	1424	2477	241-14222	81160	19674	54307	8162-311565	4689	2196	5051	876-12882
Grade													
Low	28 (11%)	541	449	409	185-2178	25615	14947	46648	4274-257344	2263	1904	1904	717-10850
High	148 (57%)	2090	653	4579	162-42523	55768	20225	67979	6322-311565	4589	2299	7255	741-72192

^†^Represents multiple histologies (see Table 1).

^‡^There are too few samples of other histotypes for analysis.

**Figure 4:**
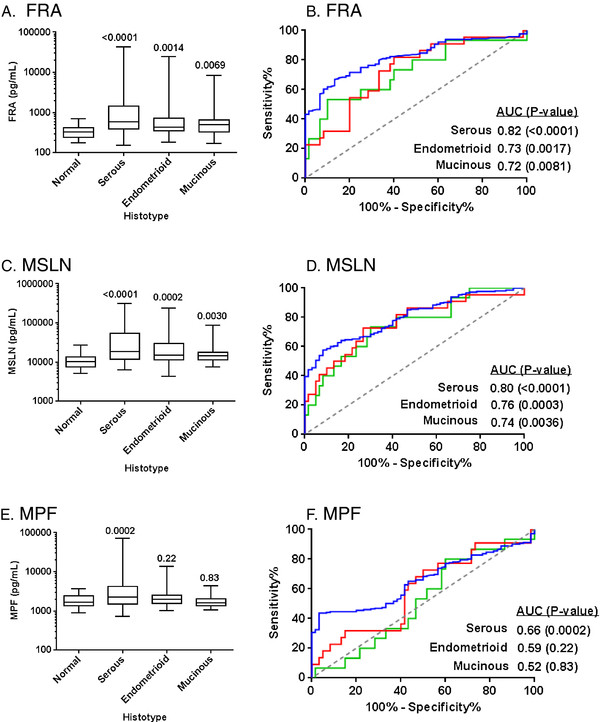
**Box plots of serum levels of A) FRA, C) MSLN and E) MPF in healthy controls and ovarian cancer patients by tumor type: serous, endometrioid and mucinous.** Boxes indicate the 25th to 75th percentiles. The horizontal lines within the boxes are the median serum levels and the whiskers indicate the minimum and maximum values. P-values indicate statistical significance of differences between each group and healthy controls. ROC curves showing the performance of **B**) FRA, **D**) MSLN and **F**) MPF serum biomarkers in discrimination of healthy controls and ovarian cancer patients by tumor type: serous (blue line); endometrioid (red line); mucinous (green line).

Relative to histotype, FRA and MSLN both showed the highest levels and most significant discrimination with normals in the serous sub-type with p-values <0.0001. Both endometrioid and mucinous sub-types could also be discerned from normal by both FRA and MSLN although to lower significance than the serous sub-type (Figure 4). ROC curve analysis resulted in AUCs ranging from 0.72-0.82 (Figure 4). MPF on the other hand was significantly elevated only in the serous histotype with an AUC of 0.66 (Figure 4). Given the relatively low representation of endometrioid (22 samples) and mucinous (15 samples) in this cohort, the results of the analysis may be skewed and further studies in these histotypes are clearly warranted. Nonetheless, it is clear from this data that all three markers – FRA, MSLN and MPF – are elevated in serous ovarian cancer.

**Figure 5:**
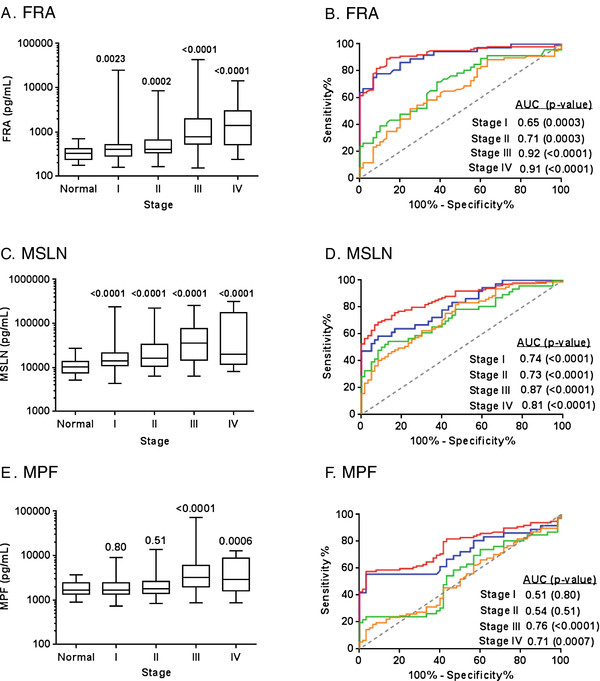
**Box plots of serum levels of A) FRA, C) MSLN and E) MPF in healthy controls and ovarian cancer patients by stage.** Boxes indicate the 25th to 75th percentiles. The horizontal lines within the boxes are the median serum levels and the whiskers indicate the minimum and maximum values. P-values indicate statistical significance of differences between each group and healthy controls. ROC curves showing the performance of **B**) FRA, **D**) MSLN and **F**) MPF serum biomarkers in discrimination of healthy controls and ovarian cancer patients by stage (I, orange line; II, green line; III, red line; IV, blue line).

**Figure 6:**
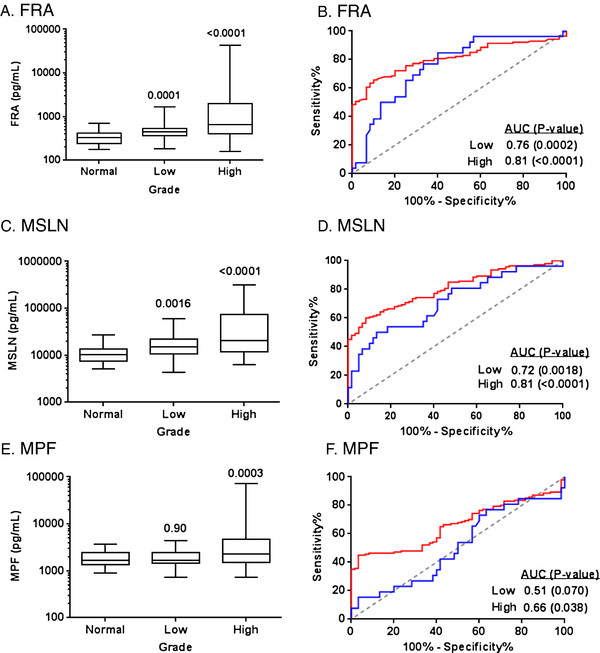
**Box plots of serum levels of A) FRA, C) MSLN and E) MPF in healthy controls and ovarian cancer patients by grade.** Boxes indicate the 25th to 75th percentiles. The horizontal lines within the boxes are the median serum levels and the whiskers indicate the minimum and maximum values. P-values indicate statistical significance of differences between each group and healthy controls. ROC curves showing the performance of **B**) FRA, **D**) MSLN and **F**) MPF serum biomarkers in discrimination of healthy controls and ovarian cancer patients by grade (low, blue line; high, red line).

The serum levels of FRA, MSLN and MPF also differed by stage (Figure 5) and grade (Figure 6) of disease. All three markers showed a significant correlation to stage of disease and significantly higher levels in high grade tumors relative to low grade tumors. While levels of FRA and MSLN were also elevated in low grade tumors relative to normal, MPF was not able to distinguish low grade tumors from normal individuals. As shown in Figures 5 and 6, FRA and MSLN were more similar in diagnostic performance, as determined by ROC analyses, than was MPF.

### Biomarker performance in serous ovarian cancer cases

Based on the data presented above and the preponderance of samples in our cohort from patients with serous ovarian cancer, a more detailed analysis of marker distributions and, importantly, comparisons to levels of CA125 and HE4 was performed on a subset of 176 serous ovarian samples.

### Correlations of biomarkers

Pairwise comparisons between FRA, MSLN, MPF, CA125 and HE4 were made through use of Pearson’s correlation coefficient (Table 3). FRA was weakly correlated with CA125, HE4 and MPF, and moderately correlated with MSLN. Like FRA, CA125 was moderately correlated with MSLN and weakly correlated with the other markers. Given the literature that MSLN interacts with CA125 and may be involved in the metastatic process, the relatively low correlation (*r*=0.53) of these two markers in serum is somewhat surprising and may reflect different mechanisms by which these two proteins enter the circulation. Further, MPF and MSLN, molecular entities derived from the same gene product, were only correlated to *r*=0.66. This is most likely a reflection of the fact that MPF is a soluble, proteolytic cleavage product of the gene product whereas MSLN is GPI-anchored and requires additional processing to be released into the circulation. Interestingly, the strongest correlation of markers was for MPF and HE4 at *r*=0.83. The relatively low correlations between these 5 serum markers suggests involvement in different and varied biological processes in ovarian cancer and suggests their usefulness in a multi-marker panel for improved discriminatory ability over CA125 alone.

**Table 3:** Pearson’s correlation coefficient between five biomarkers

	**CA125**	**HE4**	**FRA**	**MSLN**	**MPF**
CA125	1.000^****^	0.299^*^	0.316^*^	0.527^**^	0.372^*^
HE4	0.299^*^	1.000^****^	0.378^**^	0.627^**^	0.826^***^
FRA	0.316^*^	0.378^*^	1.000^****^	0.467^**^	0.233^*^
MSLN	0.527^**^	0.627^**^	0.467^**^	1.000^****^	0.659^***^
MPF	0.372^*^	0.826^***^	0.233^*^	0.659^**^	1.000^****^

Correlations were rated as follows: ****Very strong, ***Strong, **Moderate, *Weak.

### Diagnostic performance in serous histology

**Figure 7:**
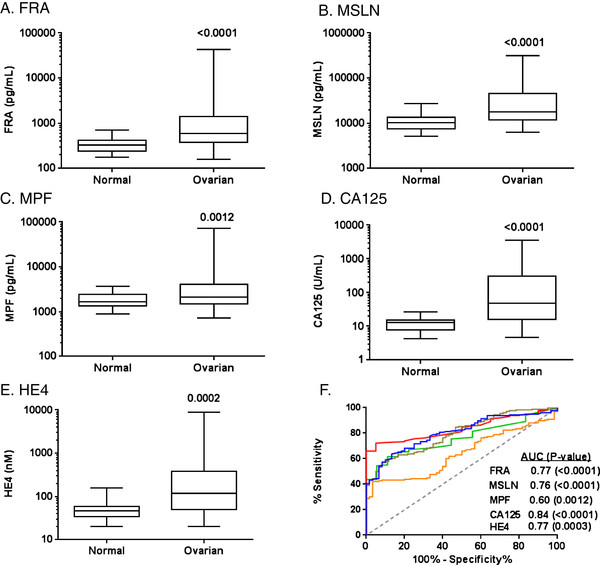
**Box plots of serum levels of A) FRA, B) MSLN C) MPF D) CA125 and E) HE4 in healthy controls and serous ovarian cancer patients.** Boxes indicate the 25th to 75th percentiles. The horizontal lines within the boxes are the median serum levels and the whiskers indicate the minimum and maximum values. P-values indicate statistical significance of differences between each group and healthy controls. **F**) ROC curves (FRA, blue line; MSLN, brown line; MPF, orange line; CA125, red line; HE4, green line) showing the performance of serum biomarkers in discrimination of healthy controls and serous ovarian cancer patients.

The serum levels of FRA, MSLN, CA125 and HE4 readily discriminated between serous ovarian cancer and control serum samples (Figure 7), with p-values of <0.0001 for FRA, MSLN and CA125, and 0.0002 and 0.0012 for HE4 and MPF, respectively. ROC analyses resulted in AUCs, in decreasing order, of 0.84 for CA125 (p<0.0001), 0.77 for FRA (p<0.0001), 0.76 for MSLN (p=<0.0001), 0.76 for HE4 (p=<0.0001), and 0.60 for MPF (p=0.0012) (Figure 7).

**Figure 8:**
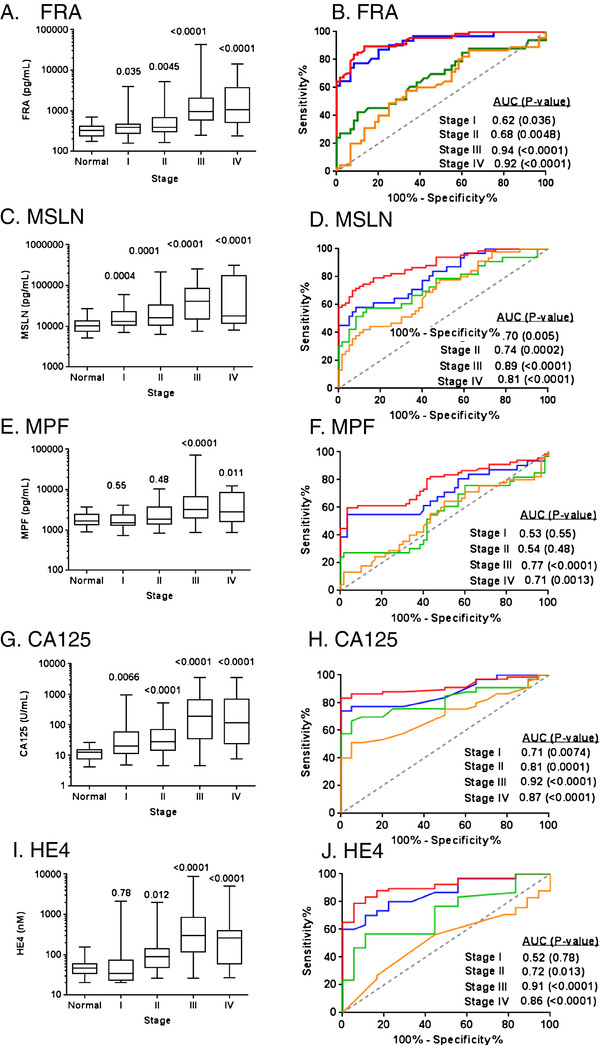
**Box plots of serum levels of A) FRA, C) MSLN, E) MPF G) CA125 and I) HE4 in healthy controls and serous ovarian cancer patients by stage.** Boxes indicate the 25th to 75th percentiles. The horizontal lines within the boxes are the median serum levels and the whiskers indicate the minimum and maximum values. P-values indicate statistical significance of differences between each group and healthy controls. ROC curves showing the performance of **B**) FRA, **D**) MSLN, **F**) MPF, **H**) CA125 and **J**) HE4 serum biomarkers in discrimination of healthy controls and serous ovarian cancer patients by stage (I, orange line; II, green line; III, red line; IV, blue line).

An assessment of each of the five biomarkers in the cohort of 176 serous ovarian cancer samples relative to stage of disease is presented in Figure 8. Each of the five markers showed clear trends of increasing levels with increasing stage of disease, although the level of significance of these changes varied widely. All markers were significantly increased in late stage disease, stages 3 and 4, relative to normal controls. CA125 and MSLN levels were shown to be significantly different from normal controls across all stages of disease.

**Figure 9:**
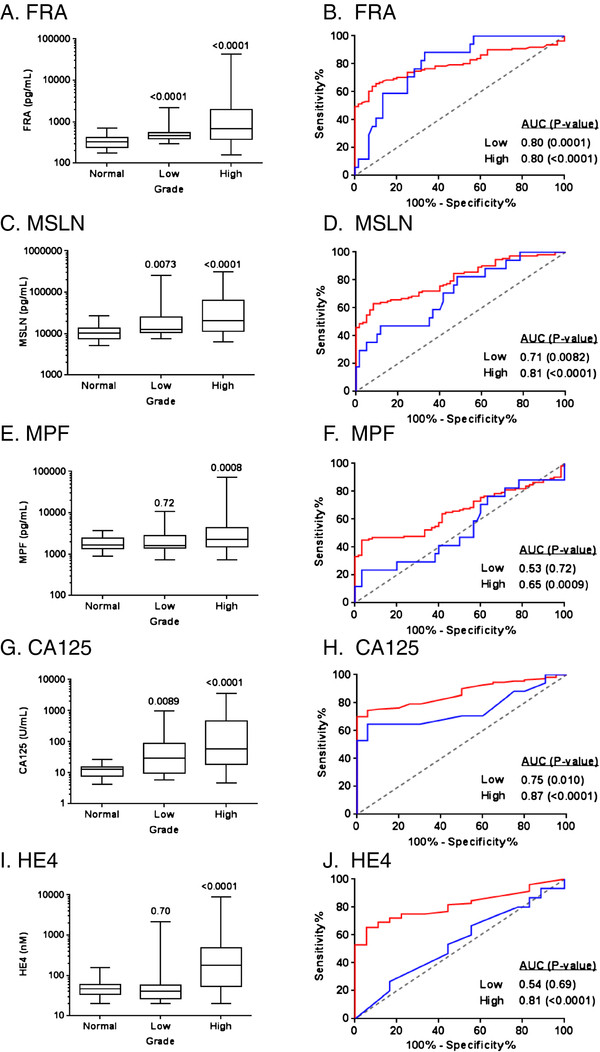
**Box plots of serum levels of A) FRA, C) MSLN and E) MPF G) CA125 and I) HE4in healthy controls and serous ovarian cancer patients by grade.** Boxes indicate the 25th to 75th percentiles. The horizontal lines within the boxes are the median serum levels and the whiskers indicate the minimum and maximum values. P-values indicate statistical significance of differences between each group and healthy controls. ROC curves showing the performance of **B**) FRA, **D**) MSLN, **F**) MPF, **H**) CA125 and **J**) HE4 serum biomarkers in discrimination of healthy controls and serous ovarian cancer patients by grade (low, blue line; high, red line).

Similarly, all five biomarkers were assessed relative to grade of disease (Figure 9). As can be seen, all markers were elevated and significantly different from normal controls in high grade disease. FRA, MSLN and CA125 were also significantly different in low grade disease. However, by ROC analysis, FRA was not able to distinguish low grade disease from normal controls (p=0.09) in this cohort. MSLN, MPF and HE4 were only able to distinguish high grade disease from normal controls, both by comparison of mean values and by ROC analysis (Figure 9).

### Multi-marker logistic regression modeling

**Figure 10:**
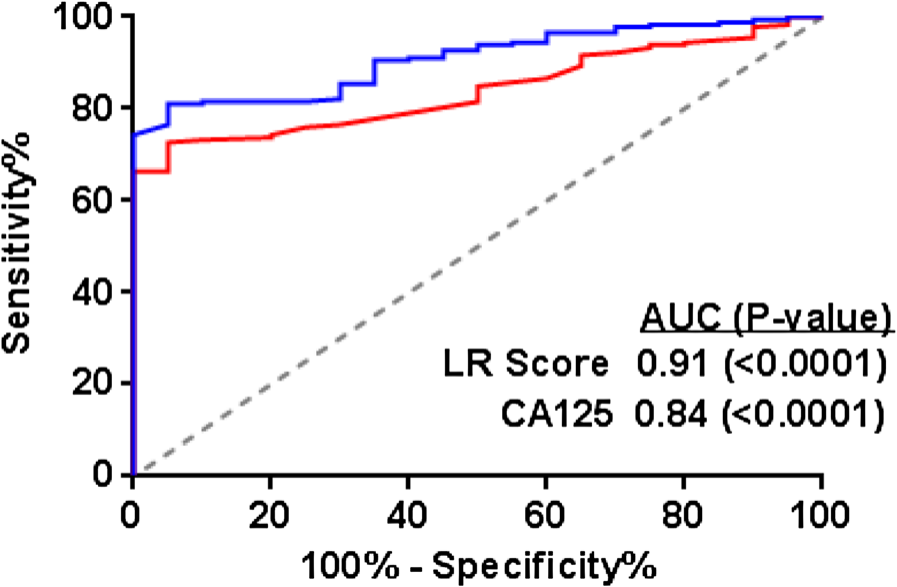
**Performance of multi-marker panels for the discrimination of ovarian cancer patients (n=176) from healthy controls (n=20).** ROC showing the performance of a four-biomarker panel, consisting of CA125, FRA, MSLN and MPF) in differentiating all ovarian cancer patients from healthy controls. The AUCs of the panel (AUC=0.91) was significantly improved over that of CA125 alone (AUC=0.84).

Given the diagnostic performance of the individual markers, and in particular the relatively weak correlation between markers, we applied logistic regression modeling in an attempt to define a combination of markers that would increase the diagnostic potential relative to the best single performing marker, CA125. In the resulting model, CA125 and MSLN were shown to increase the probability of a diagnosis of ovarian cancer as their values increased, whereas MPF showed the opposite effect. HE4 added slightly to the performance of the model, but it was not significant and therefore not included. The best model included CA125, FRA, MSLN and MPF and as can be seen in the ROC analysis presented in Figure 10, the ability of this four-marker panel to discriminate between ovarian cancer patients and healthy women was significantly improved (p=0.003) over that of CA125 alone: logistic regression (LR) AUC=0.91, p<0.0001; CA125 AUC=0.84, p<0.0001. The presented combination of markers may serve as a multi-marker panel to aid in the early diagnosis of EOC, particularly for the serous histology.

## Discussion

CA125, first introduced in the mid-1980s, remains the gold standard with respect to detection and monitoring of ovarian cancer. In recent years other markers, in particular HE4 have gained acceptance, with somewhat limited utility [64-67], and some work has been reported on combining CA125 and HE4 to further increase the diagnostic application of these serum markers [2,9,11,15]. Several other markers of ovarian cancer, including MSLN and, to a lesser extent, MPF have been described [1,15,23,30-33]. However, there are no reports to our knowledge that have performed as comprehensive an analysis of all of these markers as presented here.

FRA has been the subject of intense research as a potential therapeutic target in the last several years primarily because of its highly restricted expression profile in normal tissues [40] and high levels expression in a number of cancers of epithelial origin, including serous ovarian cancer [39-52,54-58,68,69]. Several late-stage clinical trials in ovarian cancer and non-small cell lung adenocarcinoma are presently on-going [68-70]. It is important, therefore, to develop robust assays for FRA both in tissue and in the circulation. With this in mind, the present work describes, for the first time, a specific and sensitive ECL-based assay using novel MAb reagents for the detection of FRA in serum, plasma and urine, allowing a comprehensive analysis of its diagnostic potential. Further, the potential clinical utility of serum MPF is not well documented. We therefore chose to develop a similar ECL-based assay for MPF for comparative studies not only to FRA, but to other more accepted markers of ovarian cancer – CA125, MSLN and HE4.

The described assays showed excellent limits of detection in the low pg/mL range and wide dynamic ranges up to at least 5000 pg/mL. From a practical point of view, such assays will require less in the way of repeat sample testing due to high marker levels. Importantly, FRA, MSLN and MPF were all shown to distribute equivalently between serum and plasma allowing flexibility in the choice of sample matrix. On the other hand, markers such as osteopontin are known to distribute more into the plasma fraction, restricting the sample type. FRA, MSLN and MPF were all shown to be detectable in urine samples from both healthy women and women with serous ovarian cancer. The clinical utility of MSLN measurement in urine for ovarian cancer has previously been described. In view of the ease of urine sample collection, the clinical utility of diagnostic assays assessing levels of FRA and MPF in urine is evident.

The data presented here demonstrates that for each of the markers analyzed – CA125, HE4, MSLN, MPF and FRA – there was a preferential expression in the serous histotype. Recent work from our laboratory using immunohistochemical techniques for the detection of FRA, have shown a similar preferential expression in serous carcinomas [59]. Taken together, these data support the current understanding of the origin of the various histotypes of ovarian cancer with the most common serous histotype deriving from tubal fimbriae [71]. Indeed, we recently demonstrated that FRA is highly expressed in tubal epithelium while normal ovary epithelium is devoid of FRA expression [59].

The analyzed markers showed low to moderate correlations with each other. Surprisingly, CA125 and MSLN were not highly correlated even though CA125 has been described to be the ligand for MSLN and to be involved in the metastatic process in ovarian cancer. More surprisingly, perhaps, is the moderate correlation between MSLN and MPF since these molecules should be present in a 1:1 molar ratio given the fact that they derive from the same RNA and that MPF is simply a proteolytic product of the initial gene product. However, since MSLN is a GPI-anchored protein whereas MPF is soluble, these findings, as with the correlations of the other described markers, most likely reflect a combination of the route by which the markers enter the circulation and, importantly, the clearance from the circulation. For example, while MSLN, MPF and FRA are detectable in urine, CA125 is not. Further, FRA has been shown to bind to megalin, both in the kidney and liver and, as such, is removed from circulation [72]. The glomerular filtration of at least some of these markers [32,35,73,74], as well as the potential for other clearance mechanisms, has been described previously and may be a confounding factor in the measurement and application of these markers. Ultimately, this may explain the performance of CA125 as the single best marker for ovarian cancer.

However, as described herein, combining markers with CA125 does increase the diagnostic performance of the marker panel over CA125 alone, allowing for the development of a multi-marker panel that increases the sensitivity of detection of early stage disease while retaining specificity, the ultimate goal in ovarian cancer diagnosis.

## Conclusions

CA125 remains the best single biomarker for diagnosis and monitoring of ovarian cancer. However, additional markers are sought for use independently or in combination with CA125 to improve the sensitivity for ovarian cancer detection whilst retaining specificity. The current study presents data on the utility of a novel marker, folate receptor alpha, FRA; with respect to discrimination between ovarian cancer, for example, the serous histotype, and normal controls. Further, data was presented for additional markers including MSLN and MPF and the use of these markers in a multi-marker panel that outperforms CA125 alone.

Development of additional markers for use either individually or in a panel for the diagnosis or detection of ovarian cancer, especially early stage disease, is critical. The novel ECL assays described herein provide a powerful tool for such development.

## Abbreviations

AJCC: American joint committee on cancer; AUC: Area under the curve; BMI: Body mass index; CV: Coefficient of variance; ECL: Electrochemiluminescence; EOC: Epithelial ovarian cancer; FDA: Food and drug administration; FRA: Folate receptor alpha; GPI: Glycosylphosphatidylinositol; LLOD: Lower limit of detection; MAb: Monoclonal antibody; MPF: Megakaryocyte potentiating factor; MSLN: Mesothelin; ROC: Receiver operator characteristic.

## Competing interest

The authors declare no conflicting interests in this study.

## Authors’ contributions

DJO and ES conceived and designed the experiments. PO, RH and LM performed the experiments. RPT, LMP and DJO analyzed the data. LMP and DJO wrote the paper. All authors read and approved the final manuscript.
